# The Development, Optimization and Future of Prime Editing

**DOI:** 10.3390/ijms242317045

**Published:** 2023-12-01

**Authors:** Irina O. Petrova, Svetlana A. Smirnikhina

**Affiliations:** Laboratory of Genome Editing, Research Center for Medical Genetics, Moskvorechye 1, 115478 Moscow, Russia

**Keywords:** prime editing, viral delivery, twinPE, serine integrase

## Abstract

Prime editing is a rapidly developing method of CRISPR/Cas-based genome editing. The increasing number of novel PE applications and improved versions demands constant analysis and evaluation. The present review covers the mechanism of prime editing, the optimization of the method and the possible next step in the evolution of CRISPR/Cas9-associated genome editing. The basic components of a prime editing system are a prime editor fusion protein, consisting of nickase and reverse transcriptase, and prime editing guide RNA, consisting of a protospacer, scaffold, primer binding site and reverse transcription template. Some prime editing systems include other parts, such as additional RNA molecules. All of these components were optimized to achieve better efficiency for different target organisms and/or compactization for viral delivery. Insights into prime editing mechanisms allowed us to increase the efficiency by recruiting mismatch repair inhibitors. However, the next step in prime editing evolution requires the incorporation of new mechanisms. Prime editors combined with integrases allow us to combine the precision of prime editing with the target insertion of large, several-kilobase-long DNA fragments.

## 1. Introduction

The original CRISPR genome editing relied on the RNA-guided CRISPR/Cas nuclease system, which is a natural prokaryotic system [1]. It involves the Cas protein, an RNA-guided DNA endonuclease. There are many Cas protein variants, and the most commonly used are *Streptococcus pyogenes* Cas9 (SpCas9) and *Staphylococcus aureus* Cas9 (SaCas9). A specific protospacer adjacent motif (PAM) is necessary for Cas9 binding. It should be found directly downstream of the target sequence, on the non-target strand. The PAM sequence is specific to the Cas gene and depends on the bacterial species. The SpCas9 nuclease recognizes a PAM sequence of 5′-NGG-3′. This is the least restrictive of the known PAM sequences found in nature. SaCas9 requires the 5’-NNGRRT-3’ PAM, and SaCas9 demonstrates greater activity in cell culture than SpCas9 [2].

The Cas protein needs a guide RNA molecule for target recognition. Upon the binding of the Cas–gRNA complex with the target sequence, Cas induces double-stranded breaks (DSBs), which are then repaired by either non-homologous end joining (NHEJ) or homology-directed repair (HDR) mechanisms. NHEJ is an error-prone mechanism, due to repeated cut and fix cycles, which can create arbitrary insertions and deletions, leading to frameshift mutations, premature stop codons and, eventually, gene knockout. On the other hand, HDR employs an exogenous template to generate precise DNA modifications. However, HDR is limited to actively dividing cells.

**Figure 1:**
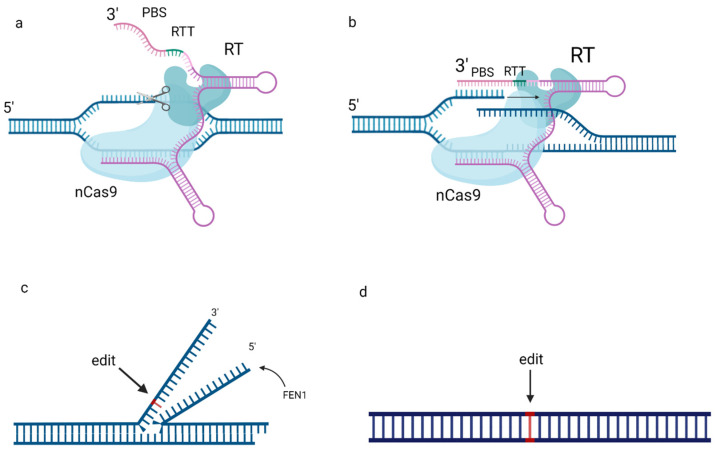
The prime editing mechanism. (**a**) Prime editor fusion protein consists of nCas9 nickase and reverse transcriptase (RT). Prime editing guide RNA includes a guide RNA fragment that binds the DNA target to expose the opposite strand, which is nicked by nCas9. The nicked fragment binds with the primer binding site (PBS) of pegRNA. (**b**) The nicked strand serves as a primer for RT, which elongates the nicked strand using a reverse transcription template (RTT) of pegRNA as a matrix. (**c**) The heteroduplex is resolved, and the unedited 5′ flap is targeted by FEN1 exonuclease. (**d**) After DNA repair, the edit is incorporated. Created with BioRender.com.

Prime editors (PEs) were developed for precise genome editing, including all types of substitutions, small insertions and deletions [3]. Prime editors use an engineered reverse transcriptase (RT) fused to Cas9 nickase (nCas9) and a prime editing guide RNA (pegRNA), which contains a guide RNA, a primer binding sequence (PBS) and a reverse transcription template (RTT) sequence, which serves as a matrix carrying the desired edit. nCas9 can be a H840A-substituted SpCas9 or N580A-substituted SaCas9 [4]. The 5′ of the pegRNA binds to the target region to expose the opposite strand, which is nicked by the nCas9 nickase and serves as a primer for the reverse transcriptase, where the pegRNA RTT serves as a matrix for elongation. Thus, the target region is modified according to the desired edit. The newly synthesized 3′ flap containing the edited sequence replaces the unedited 5′ flap sequence. The opposing unedited strand is then repaired using the edited DNA strand as a template. The 5′ flap is eliminated by structure-specific endonucleases such as FEN1, which excises 5′ flaps generated during lagging-strand DNA synthesis and long-patch base excision repair (Figure 1). The decrease in prime editing efficiency upon the inactivation of FEN1 supports this putative mechanism [5].

Five versions of the prime editor system have been characterized. PE1 is an nCas9 nickase fused to the wild-type Moloney murine leukemia virus (M-MLV) reverse transcriptase. PE2 employs an engineered pentamutant M-MLV RT that increases the editing efficiency by about three times. PE3 is a system of the PE2 fusion protein, pegRNA and an additional gRNA that targets the non-edited strand for nicking, further increasing the editing efficiency. The PE3b approach requires sgRNA sequence complementarity with the edited strand of the DNA sequence to nick the opposite strand after the editing.

DNA mismatch repair (MMR) genes strongly suppress the prime editing efficiency and promote indel formation, increasing the frequency of unintended flap rejoining outcomes. This effect was proven for *MSH2*, *MSH6*, *MLH1*, *PMS2* and *EXO1*. MMR was postulated to target a DNA heteroduplex formed by the hybridization of the reverse-transcribed 3′ DNA flap to the non-edited strand [5]. The elimination of MMR was found to create a 2–17-fold increase in PE efficiency. The proposed mechanism is the selective excision of the edited strand in the heteroduplex and subsequent repair to regenerate the original, unedited sequence [6], or, alternatively, the prevention of the annealing of the edited 3′ flap to the genomic target. Interestingly, G/C to C/G edits, which form C:C mismatches, are less frequently removed by MMR factors [7].

Unlike CRISPR/Cas9, PE should not lead to double-strand breaks. However, using qRT-PCR, the mRNA levels of cyclin-dependent kinase inhibitor 1A encoding p21 (CDKN1A) were found to be increased in PE3-edited cells. CDKN1A is involved in the repair of DSBs [8]. One of the possible reasons for DSB formation in prime editing is MMR activity, possibly via the nicking or excision of the target locus [5,7]. PE3, which creates nicks in both DNA strands and is particularly prone to MMR activation.

PE4 and PE5 prime editing systems were developed by Chen et al. [5] as PE2 and PE3 derivatives, in which the transient expression of an engineered protein (MLH1 Δ754–756 termed MLH1dn) that partially inhibits MMR enhanced the efficiency of substitution, small insertion and small deletion prime edits compared to PE2 and PE3 systems, and also improved the edit/indel ratios in MMR-proficient cell types. MMR inhibition was reported to reduce the frequency of unintended flap rejoining outcomes for reverse-transcribed 3′ DNA flaps. MLH1dn increased the PE2 efficiency in model experiments with up to a 3.2-fold improvement and the PE3 efficiency with up to a 1.2-fold improvement [5]. Reduced indel frequencies were also demonstrated. Obviously, the degree of improvement depends on the edit in question, because some edits are more affected by MMR than others. C⋅C mismatches are not efficiently repaired by MMR [7], so G/C and C/G edits are the least affected by MMR inhibition. The versions of prime editors are summarized in Table 1.

Interestingly, the installation of additional silent mutations in the edited region was proposed as a way to weaken the recognition of the heteroduplex by the MMR machinery, reducing its activity. This may explain the increased efficiency of multiple-nucleotide substitution RTs in plants, and this effect is prominent in the case of PE3 [9].

Prime editing is not independent of the replication status of the target cells. Prime editing efficiencies were superior in populations with the highest frequencies of actively cycling cells, as was shown in experiments with myoblasts and myotubes [10]. The authors supposed that DNA synthesis on edited strands or replication-dependent DNA repair processes might contribute to the increase in prime editing efficiency in replicating cells, but the exact mechanism is difficult to predict. Unlike CRISPR, PE is not based on HDR, which is active in dividing cells. Paradoxically, some level of MMR might be necessary for successful PE, because heteroduplex resolution in the final stage of the PE mechanism (see Figure 1) depends on it, and MMR is mostly active in dividing cells [11]. On the other hand, the high efficiency of MMR suppresses the PE [5]. Wang et al., in [10], used the PE2 system, and it would be interesting to compare the editing efficiencies for dividing and non-dividing cells for PE3, where a non-edited strand is nicked to shift the heteroduplex resolution by MMR towards edit incorporation. Note that MMR inhibition led to a smaller editing efficiency improvement in PE3 than in PE2 [5]. This interesting problem requires further investigation.

The adeno-associated virus (AAV) genome is flanked by two inverted terminal repeats (ITRs) that contain *cis* elements, required for replication and packaging, and the packaging-competent form of the AAV genome is ~4.7 kilobases (kb). Prime editors are 6.3 kb long, well beyond the packaging constraints of AAV. The most common delivery system for prime editors is the dual-AAV system [10,12,13]. She et al. [14] optimized the dual-AAV split-intein system for the delivery of prime editors, using inteins, protein segments that are capable of ligating the flanking exteins (external proteins) into a new protein, such as Rma or Npu. Thus, PE is split into N-terminal and C-terminal components, delivered by different vectors and reassembled in the cell. Rma split in the 1105 position of the nCas9 domain showed the best performance in vitro. A correction rate of up to 15.9% was achieved in vivo in a mouse model. Later, the dual-AAV system was optimized in vivo in a mouse model [15]. Alternatively, the dual-AAV system can involve vector genome recombination and RNA trans-splicing [13,16]. Unfortunately, the intein recombination system is only partially controllable, reducing the efficiency of dual-vector designs.

Fully viral gene-deleted adenoviral vectors, termed adenovector particles (AdVPs), can be used for the all-in-one transfer of full-length prime editing components [10]. Lentiviral vectors enable the efficient delivery of CRISPR/Cas tools in vivo [17]. All-in-one lentiviral vector systems for PE delivery have been introduced [18].

## 2. Optimization of pegRNA Design

**Figure 2:**
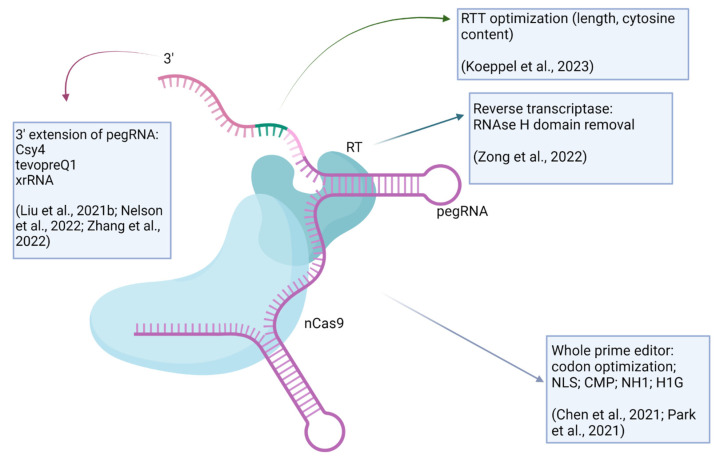
The optimization of prime editing. The 3′ extensions of pegRNA, protecting it from circularization and exonucleases, are proposed [4,33,34]. Reverse transcription template can be optimized regarding its length and nucleotide content [19]. Reverse transcriptase is optimized by removal of RNAse H domain to inhibit RNase H-directed degradation of the RNA strand in an RNA–DNA heteroduplex [35]. The entire prime editor is modified by codon optimization (using codons that are prevalent in the genetic code of the target organism) and/or inclusion of nuclear localization signal (NLS) [5], chromatin-modulating peptides (CMPs), high mobility group nucleosome binding domain 1 (HN1) and histone H1 central globular domain (H1G) [36]. Created with BioRender.com.

There is a constant demand for methods of optimization for prime editing (Figure 2). PE guide RNA design consists of four components: the single guide RNA (sgRNA), which targets a specific site to induce a nick; the primer binding site; the reverse transcription template, which includes the desired edit; and a secondary guide RNA (gRNA) to improve the editing efficiency (for PE3). The influence of the length and composition of the RT template on the editing efficiency was studied, and the results were used to develop a web application to predict prime editing insertion rates [19]. Sequences with four or more consecutive adenines in the inserted DNA (termination signal for RNA polymerase III) were 4.8-fold less expressed. The efficiency of editing depends on the effects of the insert sequence length, cytosine content and structure. Nonetheless, significant target specificity was demonstrated, making an outline of general patterns in editing efficiency challenging.

Recently developed software applications for pegRNA design are summarized in Table 2. They differ in the optimization factors for sequence design, in the Cas variants that are supported and also in the evaluation of off-target effects.

pegRNA design presents a range of opportunities for improvement. For example, the PBS is complementary to part of the gRNA spacer at the 5′ end of the pegRNA, and they can bind complementarily, causing pegRNA circularization. Liu et al. [31] fused the 20-nt Csy4 recognition site to the 3′ end of canonical pegRNA to prevent pegRNA circularization by forming a hairpin structure. The extended pegRNA outperformed the canonical pegRNA in inducing Cas9-mediated indels, increasing the efficiency up to 5.4-fold. Moreover, an additional gRNA for PE3 was fused to the extended pegRNA, enabling their co-expression in a single transcript. Meanwhile, to release the gRNA from the transcript, Liu et al. suggested fusing the reverse transcriptase with Csy4-T2A, the Csy4 RNase that selectively cleaves at the 3′ end of the Csy4 recognition site. Liu et al. [31] also modified the pegRNA scaffold to substitute the fourth uracil of consecutive uracils to cytosine, eliminating the transcription termination signal. However, the addition of Csy4-T2A enlarges the PE fusion protein, further complicating the delivery. In [32], the stem-loop aptamer MS2 was added at the 3′-terminus of the pegRNA, and N-terminus of Cas9 in the prime editor was fused with the MS2-binding protein tdMCP to ensure PE–pegRNA binding. This approach also enlarges the PE fusion protein.

Nelson et al. suggested the incorporation of RNA structures into the 3′ termini of pegRNAs to protect the 3′ extension from 3′ exonucleases, creating engineered pegRNAs (epegRNAs). A 3–4-fold increase in editing efficiency was noted. A computational tool named pegLIT was developed to identify non-interfering nucleotide linkers between pegRNAs and 3′ motifs [33] (https://peglit.liugroup.us (accessed on 19 November 2023)). Possible motifs include tevoperQ1 [33] or a viral exoribonuclease-resistant RNA motif (xrRNA) [34].

## 3. Optimization of Prime Editor

Prime editors also can be optimized. Liu et al. described a nuclear localization signal (NLS) sequence-optimized PE2 (termed PE2 *) and demonstrated its efficiency in vivo in adult mice [4]. N-terminal c-Myc NLS17 was added, as well as both a variant bipartite SV40 NLS (vBP-SV40) and SV40 NLS at the C terminus of the PE. The prime editor variant using chromatin-modulating peptides (CMPs), high mobility group nucleosome binding domain 1 (HN1) and histone H1 central globular domain (H1G) was termed CMP-PE and used as a CMP-PE3 application in mouse cells and embryos [36].

The next step was to combine several approaches for the optimization of the prime editor. PE2 was further optimized by using human codon-optimized RT, R221K N394K mutations in SpCas9, a 34-aa linker containing a bipartite SV40 NLS and an additional C-terminal c-Myc NLS, creating PEmax [5], which demonstrated better efficiency than PE2 * or CMP-PE. PEmax can be used in PE2, PE3, PE4 or PE5 methods. The combination of PE4max with epegRNAs demonstrated an increase in editing by an impressive 72 times in MMR-proficient HeLa cells. PE5max with epegRNAs demonstrated high efficiency in maize plants [37]. An enhanced plant prime editor 2 system, enpPE2, was later designed by updating the PE architecture to PEmax and expressing the engineered pegRNA with a structured motif under the control of a composite promoter [38]. A variant called KKH SaCas9, identified by molecular evolution, shows robust genome editing activity at endogenous human target sites with NNNRRT PAMs [39]. This Cas variant was also used for prime editing (SaKKHPE2 *) [4].

The Moloney murine leukemia virus reverse transcriptase was engineered by removing its ribonuclease H domain to inhibit the RNase H-directed degradation of the RNA strand in an RNA–DNA heteroduplex and incorporating a viral nucleocapsid protein with nucleic acid chaperone activity, thus creating an engineered plant prime editor (ePPE) [35].

Interestingly, the direct fusion of cell factors, such as FEN1 or T5 exonuclease, to the prime editor decreases the editing efficiency [7].

The co-delivery of p53DD, a dominant negative fragment of the p53 transcription factor, was found to greatly enhance the PE2 and PE3 efficiency in generating precise mutations in human pluripotent stem cells [40]. Recruitment of the P65 protein was found to improve the prime editing efficiency in both the PE3 and PE5 systems [7]. The reason might be that transcription factors can loosen the dense chromatin structure and allow prime editors to bind to the corresponding site, thus promoting the editing efficiency.

In effort to improve the efficiency of PE, Adikusuma et al. [41] replaced the nickase in the prime editor fusion protein with the SpCas9 nuclease, achieving higher editing efficiency at the cost of lower precision. The insertion of RT template sequences at the unmodified break sites was detected, creating unpredicted duplications. This finding undermines the precision as one of the main advantages of PE, but proves that DNA nicking is a limiting factor of PE efficiency. To improve precision of PE, H840A nCas9 was modified by the addition of another mutation (N863A) to prevent DSB formation, providing a dramatic increase in the purity of editing [42].

To assess off-target candidates for PE, a cell-based assay, named TAgmentation of Prime Editor Sequencing (TAPE-seq), was created by Kwon et al. [43]. It is based on the high-throughput sequencing of genome fragments with the incorporation of tag sequences. TAPE-seq tag is a specific sequence, which is included in the pegRNA itself. There are some limitations to this method; for example, cell-specific activity might vary between a model cell line and other cell types, causing higher off-target prediction miss rates. Secondary structures formed by tag sequences in combination with pegRNA also might cause mispredictions. Nevertheless, TAPE-seq shows high predictive power for the genome-wide off-target effects of PE2, with high validation rates and low miss rates.

One of the main problems in CRISPR/Cas-based gene editing is the requirement of a specific motif (PAM) near the target sequence for Cas9 and Cas12a proteins. Walton et al. developed the nearly PAMless SpCas9 variant using structural protein engineering [44]. After this, Qin et al. [45] combined prime editing with the versatility of prime editors (PEs) and the unconstrained PAM requirement of a SpCas9 variant (SpRY) [44], referred to as PE^SpRY^, and employed it to correct a pathogenic variant of the *Pde6b* gene in a retinitis pigmentosa mouse model in vivo. A dual-AAV vector system was used for transduction. The highest editing efficiency was up to 76.34%, whereas the conventional PE did not demonstrate any editing due to the lack of a suitable PAM. The percentage of indels was lower than 0.14%. The study did not demonstrate an increase in off-target editing, which is a major concern for PAMless editing. PAMless editing might be useful for the editing of genomic loci that do not contain a PAM sequence. On the other hand, the PAM sequence for the generally used SpCas9 (5′-NGG-3′) is quite permissive.

## 4. Next Generation of Prime Editing

Prime editing is limited mostly by the size of the pegRNA, which has to serve both as a guide for the recognition of the target sequence and as a template for editing, and also include structural motifs for nCas9 recognition. Therefore, the scope of edits that can be performed using PE is still limited.

The most widely used approach is to use two pegRNAs to overcome the size restriction for PE-based edits.

**Figure 3:**
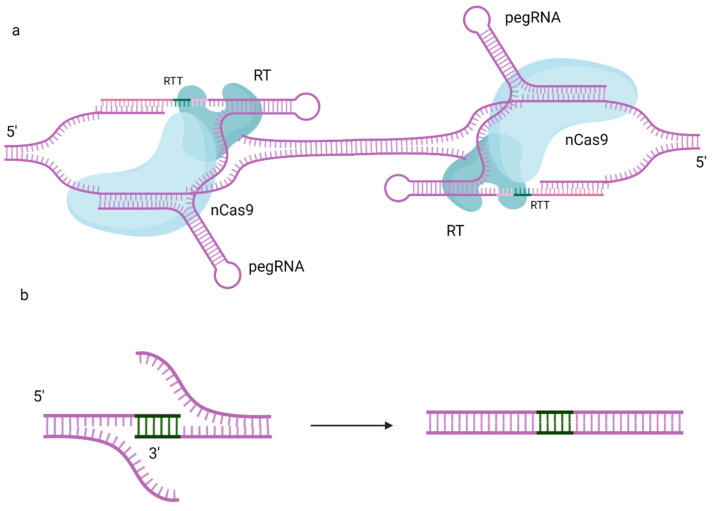
twinPE mechanism for insertion and/or deletion of small fragments using two pegRNAs. The RTT sequences of these pegRNAs are complimentary to each other (**a**), so that the edited strands create the insert, which replaces the fragment between binding sites of two pegRNAs (**b**). Created with BioRender.com.

Lin et al. [46] and Zhuang et al. [47] used dual pegRNAs in trans encoding the same edits for the forward and reverse cases for each of the DNA strands simultaneously to enhance the PE efficiency (Figure 3). Dual pegRNAs were used for large sequence (up to 861 bp) deletion, such that each pegRNA served as a gRNA for the other in bidirectional PE3 [48]. A prime editing-based method, PRIME-Del, which induces a deletion using dual pegRNAs that target opposite DNA strands [49], enables deletions up to 10 kb in length.

An alternative approach is the use of the genomic insertion mechanism of retrotransposons [50]. The pegRNA in this approach includes a second PBS in the reverse-transcribed sequence, which binds with the nicked opposite strand, allowing one to replace the fragment between two PBSs with the necessary insertion. This method, called template jumping prime editing (TJ-PE), allowed for the insertion of up to 800 bp, but in a seamless manner. Unfortunately, it requires quite large pegRNA to carry the template for insertion, so, in [50], the pegRNA was split into sgRNA and a prime editing template RNA, losing the all-in-one molecule advantage of prime editing. On the other hand, this might make the design of experiments easier, allowing for multiple combinations of target recognition sites and insert sequences.

Programmable addition via site-specific targeting elements (PASTE), which uses a nCas9 fused to both a reverse transcriptase and serine integrase, allows the insertion of long sequences (~36 kilobases) to the site of interest [51]. Serine integrases perform the site-specific recombination of small sequences of DNA called attachment (att) sites and are able to rearrange large DNA fragments combined with such sites. The addition of serine integrase to the nCas9 nickase and reverse transcriptase expands the range of PE capabilities, previously limited to single-nucleotide substitutions, deletions and insertions.

Anzalone et al. combined the use of serine integrases with twin prime editing (twinPE), using dual pegRNAs (Figure 3) [52]. A pair of pegRNAs carrying the templates for serine integrase recognition sites was proposed. In this method, the synthesized DNA sequences are not complementary to the target site, only to each other. The created complementary flaps anneal with each other, and the original sequence remains in the 5′ flaps. Recombinase recognition *attB* and *attP* sites are inserted at different genomic loci using twinPE, and the serine integrase performs the targeted integration and inversion of several-thousand-kb-long DNA fragments, thus surpassing the PE restrictions. Thus, the newly synthesized sequence replaces the original DNA sequence between the PE-induced nick sites. A similar approach, involving an engineered prime editor with enhanced editing efficiency in plants (ePPE) [39] and Cre-Lox66/Lox71 or FLP-FRT1 recombinase systems, was described as PrimeRoot [53]. PrimeRoot was able to insert fragments of up to 11.1 kb into the rice genome. It is important to note that this method is not seamless; it leads to the insertion of recombinant attL and attR sites, which might interfere with the product. Partially specific integration into native sequences that have partial sequence identity with recombinase sites, as well as large deletions and chromosome rearrangements, cannot be ruled out either [54]. Integrase-based methods depend on the activity of the integrase in question, which might provide unnecessary effects in the creation of experimental models based on the controllable activation of Cre, PhiC31 or similar enzymes. It is also worth noting that PASTE requires a donor molecule in addition to pegRNA. This donor molecule should, by necessity, be quite large, because the smaller inserts could be carried out using PE. Therefore, it complicates the delivery of the whole prime editing system to the cell, potentially reducing the efficiency.

**Figure 4:**
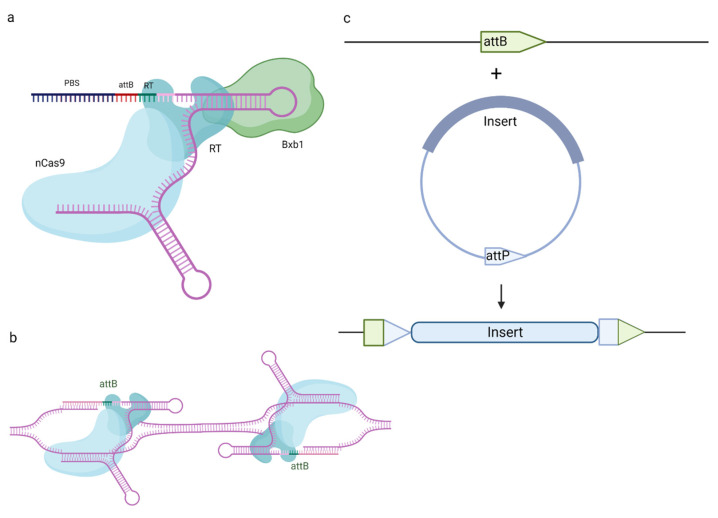
Combination of prime editing and serine integrase allows insertion of large DNA fragments. attB site can either be incorporated into reverse transcription template (**a**) or inserted by twinPE (**b**). The insert of choice carries the attP site for serine integrase (Bxb1, for example), which brings the sites together using protein–protein interactions, and carries out site-specific recombination to generate two new sites, attL and attR (**c**). Created with BioRender.com.

The first iteration of CRISPR/Cas-based genome editing was based on NHEJ and HDR with the use of exogenous donor DNA sequences, mostly in the form of ssODN. Therefore, it could be used to disrupt the sequence of choice randomly or, less effectively, to insert minor substitutions, insertions or deletions, but only in dividing cells, as HDR is active only in dividing cells. Thus, prime editing represented a major improvement, because, while still using the Cas-gRNA system for target recognition, it incorporated a novel approach to editing. Prime editing employs a reverse transcriptase to generate an edited sequence and, importantly, does not create DSBs, using an nCas nickase instead of a Cas protein. This approach allows for minor substitutions, insertions and deletions with a decreased risk of indel formation. There is still the problem of the insertion of long sequences. Prime editors combined with integrases allow us to combine the precision of prime editing with the target insertion of large, several-kilobase-long DNA fragments. Thus, integrase-based prime editing represents the next generation of gene editing (Figure 4).

The recent advancements in PE-based methods are aimed at overcoming its restriction of small-scale editing. Dual pegRNA-based solutions show distinct promise and are seamless, but are still limited regarding the scope of possible edits. Recombinase-based solutions, on the other hand, allow massive insertions but create insertions of unnecessary sites and might have off-target activity. The demand for large (hundreds and thousands of bases) insertion strands, and all the subsequent approaches, will be evaluated for this task.

## 5. Conclusions

The development of CRISPR/Cas-based gene editing is an evolutionary process, where each new, major stage is the incorporation of a new enzymatic mechanism. Prime editing was created as a combination of nCas nickase and reverse transcriptase, and PASTE followed with the addition of a serine integrase to PE. An interesting topic would be the possible next generation of CRISPR/Cas-based systems. Will it require the recruitment of some other molecular mechanism and what will it be? We might expect that other types of recombinases will be used, such as Cre or other tyrosine recombinases. On the other hand, the addition of new proteins to the editing system complicates the delivery. Even the prime editor size is beyond the carrying capacity of adeno-associated viral vectors and requires a dual-AAV intein-based system. PASTE editing requires simultaneous transfection with at least three plasmids (PE plasmid, pegRNA plasmid, Bxb1 plasmid) and a DNA donor molecule. All in all, a delivery system for PASTE and similar methods is yet to be optimized. Nevertheless, the described methods and many possible avenues for PE optimization present several opportunities for genome editing in biology and medicine, vastly expanding the applicability of CRISPR/Cas.

## Figures and Tables

**Table 1:** Versions of prime editors.

Name	Description
PE1 [3]	Original prime editor
PE2 [3]	PE1 with 5 point mutations in reverse transcriptase to increase its activity
PE3 [3]	PE2 combined with additional nicking gRNA to ensure the replacement of non-edited strand
PE4 [5]	PE2 coexpressed with MMR-inhibiting MLH1dn to improve the efficiency of editing
PE5 [5]	PE3 coexpressed with MMR-inhibiting MLH1dn

**Table 2:** Software for pegRNA design.

Application	Off-Target Effects	PAMless Cas9 Variants	Web Address	Reference
Prime Editing Design Tool	Yes	Yes	https://primeedit.nygenome.org/ (Accessed on 19 November 2023)	[20]
pegFinder	No	Yes	http://pegfinder.sidichenlab.org/ (Accessed on 19 November 2023)	[21]
PrimeDesign	Yes	No	http://primedesign.pinellolab.org/ (Accessed on 19 November 2023)	[22]
CRISPRseek	Yes	No	https://bioconductor.org/packages/release/bioc/html/CRISPRseek.html (Accessed on 19 November 2023)	[23]
multicrispr	Yes	No	https://www.bioconductor.org/packages/release/bioc/html/multicrispr.html (Accessed on 19 November 2023)	[24]
Prime Induced Nucleotide Engineering Creator of New Edits (PINECONE)	Yes	No	https://github.com/xiaowanglab/PINE-CONE (Accessed on 19 November 2023)	[25]
PE-Designer	Yes	Yes	http://www.rgenome.net/pe-designer/ (Accessed on 19 November 2023)	[26]
PnB designer	No	No	https://fgcz-shiny.uzh.ch/PnBDesigner/ (Accessed on 19 November 2023)	[27]
pegIT	Yes	Yes	https://pegit.giehmlab.dk/ (Accessed on 19 November 2023)	[28]
Easy-Prime	Yes	No	http://easy-prime.cc/ (Accessed on 19 November 2023)	[29]
PlantPegDesigner	No	Yes	https://github.com/JinShuai001/PlantPegDesigner (Accessed on 19 November 2023)	[30]
